# Well-being of professional older adults’ caregivers in Alberta’s assisted living and long-term care facilities: a cross-sectional study

**DOI:** 10.1186/s12877-023-03801-9

**Published:** 2023-02-09

**Authors:** Oluwagbohunmi A. Awosoga, Adesola Christiana Odole, Ogochukwu Kelechi Onyeso, Jon Doan, Christina Nord, Ifeoma Blessing Nwosu, Claudia Steinke, Joshua O. Ojo, Ezinne Chika Ekediegwu, Sheli Murphy

**Affiliations:** 1 Faculty of Health Sciences, University of Lethbridge, Lethbridge, AB Canada; 2 Department of Physiotherapy, Faculty of Clinical Sciences, College of Medicine, University of Ibadan, Ibadan, Oyo Nigeria; 3 Emerging Researchers and Professionals in Ageing-African Network, Abuja, Nigeria; 4 Faculty of Art and Sciences, University of Lethbridge, Lethbridge, AB Canada; 5 Department of Medical Rehabilitation, Faculty of Health Sciences and Technology, College of Health Sciences, Nnamdi Azikiwe University, Awka, Anambra Nigeria; 6 Rural Health, Professional Practice, Research and Libraries, Covenant Care, Edmonton, AB Canada

**Keywords:** Absenteeism, Continuing care, Health status, Job stress, Nursing home, Mental health, Physical health, Quality of life, Turnover

## Abstract

**Background:**

For the care need of older adults, long-term care (LTC) and assisted living (AL) facilities are expanding in Alberta, but little is known about the caregivers’ well-being. The purpose of the study was to investigate the physical health conditions, mental and emotional health (MEH), health behaviour, stress levels, quality of life (QOL), and turnover and absenteeism (TAA) among professional caregivers in Alberta’s LTC and AL facilities.

**Methods:**

This cross-sectional survey involved 933 conveniently selected caregivers working in Alberta’s LTC and AL facilities. Standardised questions were selected from the Canadian Community Health Survey, Patient Health Questionnaire-9, and Short Form-36 QOL survey revalidated and administered to the participants. The new questionnaire was used to assess the caregivers’ general health condition (GHC), physical health, health behaviour, stress level, QOL, and TAA. Data were analysed using descriptive statistics, Cronbach alpha, Pearson’s correlation, one-way analysis of variance, and multiple linear regression.

**Results:**

Of 1385 surveys sent to 39 facilities, 933 valid responses were received (response rate = 67.4%). The majority of the caregivers were females (90.8%) who were ≥ 35 years (73.6%), worked between 20 to 40 h weekly (67.3%), and were satisfied with their GHC (68.1%). The Registered Nurses had better GHC (mean difference [*MD*] = 0.18, *p* = 0.004) and higher TAA than the Health Care Aides (*MD* = 0.24, *p* = 0.005). There were correlations between caregivers’ TAA and each of MEH (*r* = 0.398), QOL (*r* = 0.308), and stress (*r* = 0.251); *p* < 0.001. The most significant predictors of TAA were the propensity to quit a workplace or the profession, illness, job stress, and work-related injury, *F* (5, 551) = 76.62, *p* < 0.001, adjusted R^2^ = 0.998.

**Conclusion:**

Reducing the caregivers’ job stressors such as work overload, inflexible schedule, and poor remuneration, and improving their quality of life, health behaviour, and mental, emotional, and physical health conditions may increase their job satisfaction and reduce turnover and absenteeism.

**Supplementary Information:**

The online version contains supplementary material available at 10.1186/s12877-023-03801-9.

## Background

The population of Canadian older adults is increasing and has been projected to reach between 9.9 and 10.9 million people by 2036 [1]. With the surge in the population of Canadian older adults (65 years and above), there is a corresponding increase in the health and psychosocial needs of this population [2]. Canada has a policy for universal health coverage for citizens including older adults who may require permanent residency in a continuing care facility [3]. Continuing care includes home care, assisted living (AL), long-term care (LTC), hospice, and end-of-life care [4]. There are provincial differences in continuing care policies across Canada [3], within the province of Alberta, continuing care systems provide older adults with health and social care to support their independence and good quality of life (QOL) [5].

The services rendered by LTC, and AL facilities fulfil a growing need for care of the older adult population in Canada [6]. The present study focused on professional paid caregivers attending to older adults in AL and LTC facilities. In some jurisdictions, AL and LTC facilities are referred to as nursing homes [2], but there could be slight differences in the meanings of these terms indicating the levels of care [5]. Nursing homes are used by people who do not need to be in a hospital but cannot be cared for at home [2]. According to Government sources [4, 5], AL facilities provide 24-hour accommodation and personal care support including onsite nursing and rehabilitation therapy for residents ageing in place. In addition, the LTC facilities offer individuals with complex, unpredictable medical needs 24-hour onsite health and person-centred care [5]. Continuing care is often provided by registered nurses (RN), licenced practical nurses (LPN), health care aides (HCA), and other healthcare professionals depending on needs [5]. Some organisations run both AL and LTC services in the same or separate facilities and caregivers can be switched between residents and facilities [5, 7, 8]. The work expectations and remuneration of AL and LTC caregivers are similar [9].

Canada had undertaken several initiatives to address the challenges of the aging population. For over two decades, AL facilities have been expanding to bridge the continuum of care between home living and provincially regulated LTC and AL facilities [8, 10]. Worrisomely, the needs of the LTC and AL caregivers were rarely addressed in these initiatives [3, 9]. For instance, the Action for Older Adults Report [6] did not discuss the growing staffing shortfall in AL and LTC facilities, nor the causes of organisational turnover and absenteeism (TAA) such as overwork, underpay, poor job satisfaction, and burnout [11–13]. Similarly, the National Seniors Strategy did not address any of the challenges associated with TAA among the continuing care workforce [14]. There was a paucity of data on the well-being across designations of continuing caregivers in Alberta and how it associates with TAA. This study was a large mixed-methods research designed to address these gaps; the qualitative arm of the study is published [15].

Canadian LTC and AL facilities continue to experience workforce shortages [12, 16]. Before the first wave of the Covid-19 pandemic, over 25% of Canadian continuing-care facilities had reported critical shortages in their workforce, but the figure doubled during the pandemic [2]. Specifically, in 2020, 71% of LTC facilities reported an increase in absenteeism and 50% reported critical shortages of RN, LPN, and HCA, which had an impact on the quality of resident care and caregiver well-being [17]. A pre-pandemic study estimated that there would be a shortage of 4,606 RNs in Alberta by 2023 [18]. However, the Covid-19 pandemic led to an unprecedented caregiver shortfall in the province [17]. The pre-pandemic turnover rate was associated in part with managerial incompetency, inefficient organisational policies, low wages, caregivers’ burnout, distress, and ill health [7, 9, 11, 19–21]. Canadian Institute for Health Information [19] reported that 32% of caregivers who provided more than 21 hours of care per week experienced personal health distress as a direct result of their job. Caregiving is a very demanding career, and understanding these demands has the potential to illuminate strategies to increase the quality-of-care clients receive and the productivity of caregivers while also improving QOL amongst caregivers and decreasing direct and indirect demands on the healthcare system [22].

Our study was grounded in the “happy-productive worker” theory [23], which supports the notion that efficient management of caregivers leads to effective workforce utilisation, maximisation of their scope of practice, and the collective ability for optimum patient care. Kemp et al. [24] opined that when caregivers are consistent, healthy, and well, they can provide a better quality of care to their patients. Similarly, Desimini [25] reported that there was a strong association between the QOL of LTC caregivers and the quality of care they gave. Thus, investigating the QOL and health status of LTC and AL caregivers and the implications for continuing care outcomes is a valuable endeavour.

Therefore, the current study investigated the general health condition (GHC), physical health, mental and emotional health (MEH), stress, QOL, health behaviour, and TAA among paid older adults’ caregivers working in LTC and AL facilities in Alberta. The research questions were: (a) What are the caregivers’ levels of physical health, MEH, stress, QOL, and health behaviour? (b) Is there any significant difference in physical health, MEH, stress, QOL, and health behaviour across caregiver designations? (c) Is there any significant correlation between caregivers’ physical health, MEH, stress level, QOL, and health behaviour? (d) What factors can best predict physical health, MEH, stress, QOL, health behaviour, and TAA among caregivers?

## Methods

### Study design and setting

We conducted a cross-sectional study of the physical, emotional, and mental health status of paid professional caregivers in LTC and AL facilities in Alberta, Canada between June 2017, and October 2019. There were 324 publicly funded continuing care facilities across the five Alberta Health Services (AHS) Zones: North (55), Edmonton (84), Central (73), Calgary (64), and South (48) [8]. We used electronic random number generation to select 50 facilities from the list of 324 facilities. An invite/permission letter was sent to each of the 50 selected facilities requesting for their centre to be involved in the study, however, only 39 facilities responded and were involved in the study. Ethical approvals for the study were obtained from the Human Subject Research Review, University of Lethbridge (Study#1913, REB#Pro00072081), and the Health Research Ethics Board of the University of Alberta (RA83256). All the eligible caregivers within each facility signed an individual informed consent form before partaking in the study. Participants were informed of their right to withdraw at any point in the study. The approved protocol, participants’ privacy, and confidentiality of data were strictly adhered to.

### Participants and eligibility criteria

The survey participants were professional caregivers working in 39 publicly funded LTC and AL facilities in Alberta. Participant inclusion criteria were: (a) being a casual, part- or full-time caregiver in any of the selected continuing care facilities, (b) caring for older adults, (c) having at least an elementary school education, and (d) willingness and ability to complete the survey independently.

### Sample size determination

The sample size was calculated using the G*Power 3.1.9.4 software. A sample of 436 participants was appropriate for one-way analysis of variance (ANOVA) given a moderate effect size of 0.2, error of probability = 0.05, and power = 95.0%.

### Research instrument

This study adopted questions from existing standardised instruments: Canadian Community Health Survey [26], Patient Health Questionnaire-9 [27], and the SF-36 QOL survey [28]. The draft questionnaire was structured into seven tentative domains (Parts A to G) and sent to a three-man panel of experts who completed the face and content validation through the Delphi method of email exchanges [29]. The validated instrument was pilot tested for psychometric properties among 50 conveniently selected caregivers working within the AHS South Zone, who were excluded from the main survey. The result of the pilot testing did not necessitate further modification of the questionnaire.

We computed the reliability and internal consistency of each domain of the survey instrument, the scores showed that the instrument was valid and reliable: inter-class correlation coefficient (and Cronbach’s alpha) of physical health = 0.83(0.348), health condition = 0.83(0.230), MEH = 0.93(0.303), stress = 0.85(0.360), QOL = 0.90(0.444), health behaviour = 0.82(0.265), TAA = 0.65(0.295), and overall = 0.96(0.217). We also completed an exploratory factor analysis (Maximum Likelihood with Varimax-orthogonal rotation) to confirm the domains in which individual items of the instrument fit. After the issues of communality and multicollinearity were fixed, the extracted variances assessed on a rotated factor matrix showed the questionnaire items belonging to seven distinct domains labelled parts A to G.

Parts A (16 items) and B (10 items) collected information on caregivers’ physical and general health conditions, respectively. Part C (31 items) assessed caregivers’ MEH, Part D (10 items) gathered information on stressors, and Part E (11 items) assessed the QOL. Part F (13 items) obtained data on the caregivers’ health behaviour and Part G (4 items) collected information on TAA. The last Sect. (8 items) collected the sociodemographic characteristics of the participants, such as age, gender, appointment type, designation, shift duty, weekly workload (hours), years of practice, and highest educational qualification. Parts A to G were 5-point Likert scales. The sociodemographic variables were nominal or ordinal data.

### Procedure for data collection

The first author scheduled a meeting with facility managers to acquaint them with the research objectives, the significance of the study, and the data collection procedure at least one week before the commencement of the study. After the meeting, we shared the study fact sheet posters and flyers, and letters of invitation with the potential participants to maximise the response rate. One week later, we visited each facility and delivered print copies of the survey package including the participant information sheet, informed consent form, questionnaire, and a brown envelope to all eligible and willing participants.

We recruited participants from each facility during the morning, afternoon, and night shifts for five consecutive days via convenience sampling. Participants were instructed to read and endorse the informed consent form before proceeding to answer the questionnaire. The questionnaire was self-administered. However, the first author’s telephone number and email address were boldly printed at the top of each page in case the caregivers needed further information or clarification. For confidentiality, we instructed the participants to seal their completed survey in a brown envelope and submit it in a secured box provided at the staff lounge. The first author visited each facility at the end of two months of data collection to retrieve the submitted survey. The study duration in each of the five AHS zones was approximately six months, which provided a long-term view of the activities across the study locations.

### Variables

The primary outcome variables were health condition, physical health, MEH, stress, QOL, health behaviour, and TAA, obtained on a 5-point Likert scale: 1 = lowest to 5 = highest score. Negatively worded items were reverse-coded before analysis. Each primary outcome was normally distributed. Therefore, the data were treated as ordinal continuous variables, applying relevant parametric univariate, bivariate, and multivariate analyses [30].

### Data analysis

The responses were collated in an electronic spreadsheet and analysed using the Statistical Package for Social Sciences (SPSS) software (version 24). Descriptive statistics were summarised using frequency (percentage) and mean (standard deviation). For the inferential analyses, the dataset was cleaned and diagnosed for relevant parametric test assumptions: missing values, univariate and multivariate outliers, normality, sphericity, linearity, and multicollinearity were fixed [31]. One-way analysis of variance (ANOVA) and Tukey’s post hoc test was used to test for significant differences in health status, stress and QOL across the caregiver designations. The correlations among the primary outcome variables were completed using Pearson’s correlation coefficient. Multiple linear regression was used to determine elements of health status, working conditions, stress, and QOL that best predict each of the primary outcome variables. The alpha level was set at 0.05.

## Results

### Demographic characteristics

Of the 1,385 questionnaires administered, we received 933 responses – accounting for a 67.4% response rate. The participants were Registered Nurses (RN) 13.2%, Licenced Practical Nurses (LPN) 12.4%, Health Care Aides (HCA) 58.1%, and Allied Health Workers (AHW) 16.3% working in LTC and AL facilities across the Alberta province. The AHW were administrative staff, chaplains, cooks, dietitians, housekeepers, laboratory scientists, maintenance officers, environmentalists, occupational therapists, pharmacists, physiotherapists, recreational therapists, social workers, and therapy assistants. Of the 933 participants that completed the survey, 122 (13.1%) were from five facilities in the Calgary zone, 207 (22.2%) from fourteen facilities in the Central zone, 172 (18.4%) from three facilities in the Edmonton zone, 50 (5.4%) from three facilities in the North zone, and 382 (40.9%) from fourteen facilities in the South zone. The majority (72.5%) of the participants reported English as their first language.

Table 1 shows the participants’ demographics. Most of the participants were females (90.8%), aged 35 years and above (73.6%), who had a college diploma, bachelor, or graduate degree as their highest educational attainment (73.7%). Many of the caregivers (64.0%) had five years or longer work experience, 59.5% worked rotational (day, evening, and night) shifts, while only 32.0% worked regular day shifts. In addition, 67.3% worked on average 20 to 40 h per week of which 39.5% were part-time staff including 39.2% who were on rotational shift duty. The proportion of caregivers who worked overtime (41 to 60 h) was more among RNs (31.3%) than in other designations (Table 2).

**Table 1:** Demographic characteristics of the caregivers (*n* = 933)

Variables	Total (*%*)
**Age range (years)**	
18 – 25	74 (8.1)
26 – 34	168 (18.3)
35 – 44	228 (24.9)
45 – 54	224 (24.4)
55 and above	223 (24.3)
**Gender**	
Male	83 (9.1)
Female	833 (90.8)
Non-Binary	1 (0.1)
**Type of shift duty**	
Day	293 (32.0)
Evening	49 (5.3)
Night	29 (3.2)
Rotational (Day, Evening, and Night)	546 (59.5)
**Hours worked per week**	
< 20	62 (6.8)
20 – 40	615 (67.3)
41 – 60	205 (22.4)
> 60	32 (3.5)
**Nature of appointment**	
Full-time	328 (35.2)
Part-time	494 (53.1)
Casual	109 (11.7)
**Years of Experience**	
0 – 2	129 (14.1)
2 – 5	201 (22.0)
5– 10	218 (23.9)
10 – 20	218 (23.9)
> 20	148 (16.1)
**Education**	
Less than High School Diploma	27 (3.0)
High School Diploma	161 (17.6)
College Diploma	421 (46.1)
Bachelor/Graduate Degree	252 (27.6)
Others	52 (5.7)
**Designation**	
Registered Nurse	121 (13.2)
Licensed Practical Nurse	114 (12.4)
Health Care Aide	534 (58.1)
Allied Health Worker	150 (16.3)

*n* < 933 for some variables because of missing data

**Table 2:** Crosstabulation of workload, appointment type, and duty schedule

Parameter	Less than 20 h *f* (%)	20–40 h *f* (%)	41–60 h *f* (%)	More than 60 h *f* (%)	Total *f* (%)
**Nature of appointment**					
Full-time job	1 (0.3)	189 (58.5)	119 (36.8)	14 (4.4)	323 (100)
Part-time job	32 (6.6)	359 (74.5)	76 (15.8)	15 (3.1)	482 (100)
Casual job	29 (27.9)	65 (62.5)	8 (7.7)	2 (1.9)	104 (100)
Total	62 (6.8)	613 (67.4)	203 (22.3)	31 (3.5)	909 (100)
**Type of shift duty**					
Day	19 (6.5)	193 (66.1)	69 (23.6)	11 (3.8)	296 (100)
Evening	5 (10.2)	36 (73.5)	5 (10.2)	3 (6.1)	49 (100)
Night	2 (6.9)	19 (65.5)	8 (27.6)	0 (0.0)	29 (100)
Rotational (all shifts)	36 (6.6)	366 (67.4)	123 (22.7)	18 (3.3)	543 (100)
Total	62 (6.8)	614 (67.3)	205 (22.5)	32 (3.4)	913 (100)
**Designation**					
Registered Nurse	14 (11.9)	63 (53.4)	37 (31.3)	4 (3.4)	118 (100)
Licensed Practical Nurse	4 (3.7)	73 (67.0)	28 (25.6)	4 (3.7)	109 (100)
Health Care Aide	31 (5.9)	353 (67.5)	118 (22.6)	21 (4.0)	523 (100)
Allied Health Worker	12 (8.0)	113 (75.3)	22 (14.7)	3 (2.0)	150 (100)
Total	61 (6.8)	602 (66.9)	205 (22.8)	32 (3.5)	900 (100)

Participants with missing data for work volume, nature of appointment, and workload were excluded from the analysis

### Physical and general health condition of caregivers

On average, 924 caregivers of 933 provided answers to the questions probing their physical health. The majority (77.4%) of caregivers reported that their health was at least in good condition (28.6% in very good condition and 48.8% in good condition). Only 2.0 and 0.3% reported that they were in poor and very poor physical condition, respectively, as compared with other people of their age. A little above half (59.6%) of caregivers felt a lot of energy, 13.3% felt otherwise while 27.2% were neutral. Nonetheless, 58.1% were expectant of improved health, 11.1% were satisfied with their health, and 30.8% were indifferent. Three-quarters were free from chronic diseases, while 17.3% had a chronic disease, and the rest were unsure of their status (7.7%).

Many caregivers (63.3%) reported that they were as healthy as any other person they knew, but 13.6% reported that they got sick more frequently than their peers do, and 17.4% were undecided about their health compared to others. Seventy-seven percent of caregivers were satisfied with their overall health conditions as compared to 11.8% of the caregivers that were not. We further assessed the physical health condition of caregivers based on body systems, 68.1% of caregivers reported an incidence of work-related fatigue or low energy, and 49.3% of caregivers experienced breathlessness on slight exertion at work.

### Mental and emotional health, stress level, and quality of life

Caregivers reported that they sometimes experienced mental and emotional health issues. The tabulation of the participants’ perception of their mental health in the last six months was presented in Supplementary File 1. The following were some of the significant responses from caregivers: At least 73.4% of caregivers reported having a high self-esteem or feeling happy with themselves and therefore considering themselves as happy people, 70.5% reported feeling excited to be alive when they wake up in the morning, 78.3% agreed that they have a good level of motivation, and 79.0% caregivers reported that they experienced enjoyment and fulfilment in their work. Similarly, 81.3% of caregivers reported feeling a sense of belongingness in their workplace.

Moreover, caregivers were asked to evaluate their stress levels in relation to their families, work, relationships, finances, and in general (Supplementary File 1). Most caregivers reported having at least low, medium, high, or very high-stress levels with regard to their families (83.2%), work (93.8%), health (77.0%), finances (84.5%), and coping with daily problems (80.9%). Additionally, only 23.4% of caregivers reported being stressed with school, and 51.3% reported no stressful sex lives.

The self-reported QOL of caregivers was presented in Supplementary File 1. The majority of caregivers (77.0%) reported being at least satisfied with their life as a whole. Also, 79.8% reported being satisfied with their personal lives. Only 9.3% were unhappy with their romantic lives, and 77.7% and 71.5%, respectively reported being at least satisfied with their jobs and actual accomplishments.

### Health behaviour, absenteeism, and turnover

Regarding caregivers’ health behaviour, including dieting, exercising, routine medical check-ups, alcohol intake, and smoking, at least 90.5% of the caregivers reported having a healthy diet. This included 37.2% of those who sometimes have healthy diets, 43.6% of those who regularly had healthy diets, and 9.7% of those who persistently ensure a healthy diet. In addition, 75.0% of caregivers indicated that they followed the Canadian Food Guide (7–10 servings of vegetables and fruit, 2–3 servings of meat, and 2 servings of milk). Of the participants that reported having a healthy diet, 85.7% indicated that they ate diets high in fibre, while 4.5% reported that they frequently ate fatty foods. A majority (90.3%) of the caregivers reported that they drink adequate amounts of fluids (1/2 oz per pound body weight). Three-quarters of the caregivers reported that they never smoked, and 44.8% reported that they never consumed alcohol.

Furthermore, 62.9% of the caregivers reported engaging in exercise at least three times a week for at least 20 min each time, and 35.5% reported that they did not have enough sleep at night (7–8 h of sleep). Only 19.2% reported never or infrequently visiting their doctors for medical reviews. Also 7.5, and 19.2%, respectively had never or infrequently examined themselves for warning signs of cancer (for example, breast or prostate). A whopping 97.5% of the caregivers reported that they are cautious of acute health problems such as colds, and musculoskeletal injuries.

In the last six months before taking the survey, only 16.5% of the caregivers reported that they were absent for six days or more due to ill health, and 5.9% reported that they were absent due to work-related injuries. At least 46.5% of the caregivers indicated an intention to quit their profession (professional turnover), and 51.1% reported that they contemplated changing their employment (organisational turnover).

### Inferential analysis

The ANOVA results in Table 3 showed no significant difference in the primary outcomes across designations, except GHC (*F* [3, 915] = 4.347, *p* = 0.005) and TAA (*F* [3, 915]  = 4.009, *p* = 0.008). The post hoc analyses showed that Registered Nurses had better health conditions (mean difference [*MD*] = 0.18, *p* = 0.004) and higher TAA than the Health Care Aides (*MD* = 0.24, *p* = 0.005). This shows that it is more difficult to retain Registered Nurses than Health Care Aides in low-income jobs.

**Table 3:** ANOVA: Differences in health statuses, stress level, quality of life, health behaviour, turnover, and absenteeism across caregiver designations

Parameter	N	Mean (SD)	*F*-statistic	*p*-value	Ƞ^2^
**Physical Health**			1.822	0.142	0.01
Registered Nurse	121	3.69 (0.64)			
Licensed Practical Nurse	114	3.59 (0.74)			
Health Care Aide	534	3.54 (0.70)			
Allied Health Worker	150	3.59 (0.60)			
**General Health Condition**			4.347	0.005*	0.01
Registered Nurse	121	4.06 (0.44)			
Licenced Practical Nurse	114	3.92 (0.54)			
Health Care Aide	534	3.87 (0.59)			
Allied Health Worker	150	3.97 (0.45)			
**Mental and Emotional Health**			2.136	0.094	0.01
Registered Nurse	121	3.67 (0.58)			
Licenced Practical Nurse	114	3.49 (0.58)			
Health Care Aide	534	3.56 (0.59)			
Allied Health Worker	150	3.53 (0.53)			
**Stress Level**			1.137	0.333	< 0.01
Registered Nurse	121	3.87 (0.59)			
Licenced Practical Nurse	114	3.78 (0.59)			
Health Care Aide	534	3.77 (0.72)			
Allied Health Worker	150	3.73 (0.55)			
**Quality of Life**			0.814	0.486	< 0.01
Registered Nurse	121	3.99 (0.65)			
Licenced Practical Nurse	114	3.89 (0.61)			
Health Care Aide	534	3.93 (0.66)			
Allied Health Worker	150	3.88 (0.66)			
**Health Behaviour**			2.462	0.061	0.01
Registered Nurse	121	3.42 (0.54)			
Licenced Practical Nurse	114	3.33 (0.53)			
Health Care Aide	534	3.27 (0.61)			
Allied Health Worker	150	3.28 (0.52)			
**Turnover and Absenteeism**			4.009	0.008*	0.01
Registered Nurse	121	4.52 (0.66)			
Licenced Practical Nurse	114	4.38 (0.75)			
Health Care Aide	534	4.28 (0.86)			
Allied Health Worker	150	4.45 (0.67)			

^*^F-statistic was significant at *p* < 0.05 level (2-tailed). Mean (range) = 1–5. Effect sizes (Eta squared) Ƞ^2^; 0.01 = small, 0.06 = medium, 0.14 = large, and 0.21 = much large

Pearson’s correlation results (Table 4) showed that there was a strong association between the physical health of caregivers and their GHC, MEH, and QOL. The results also indicated a moderate correlation between physical health and stress level, health behaviour, and TAA. Remarkably, TAA had an inverse correlation with caregivers’ QOL (*r* = -0.308, *p* < 0.001), as well as their MEH (*r* = -0.398, *p* < 0.001). The strongest positive correlation was between QOL and MEH (*r* = 0.660, *p* < 0.001).

**Table 4:** Pearson’s correlations coefficients

Variable	General health condition	Mental and emotional health	Stress level	Quality of life	Health behaviour	Turnover and absenteeism
Physical health	0.626**	0.606**	0.398**	0.491**	0.387**	0.278**
General health condition	-	0.541**	0.394**	0.382**	0.306**	0.269**
Mental and emotional health	-	-	0.560**	0.660**	0.405**	-0.398**
Stress level	-	-	-	0.578**	0.349**	0.251**
Quality of life	-	-	-	-	0.434**	-0.308**
Health behaviour	-	-	-	-	-	0.167**

^**^Pearson correlation (*r*) was significant at *p* < 0.001 (2-tailed)

A forward stepwise multiple linear regression was completed to determine measures of well-being and work-related factors that could best predict the study outcomes (Table 5). The models were well fit with at least 91% variance explained for each domain (adjusted R^2^ ≥ 0.91, *p* < 0.001). Workload, job stress, and workplace injury were significant predictors of MEH, stress level, QOL, and TAA (*p* < 0.01). Specifically, the most significant predictors of TAA were the propensity to quit a workplace or the profession, illness, work stress, and work-related injury, *F* (5, 551) = 76.62, *p* < 0.001, and adjusted R^2^ = 0.998 (Table 5, Model 7).

**Table 5:** Multiple linear regression models for predictors of caregivers’ well-being

(Model) DV	Determinants
**(1). Physical health**	(Constant), overall satisfaction with one’s health (β = 0.285, *p* < 0.001), feeling physical pain (β = 0.285, *p* < 0.001), feeling as healthy as others (β = 0.285, *p* < 0.001), feeling of getting sick more easily than others (β = 0.285, *p* < 0.001), being free of chronic disease (β = 0.285, *p* < 0.001), feeling a lot of energy (β = 0.155, *p* < 0.001), feeling good about one’s physical appearance (β = 0.152, *p* < 0.001), feeling limited by physical health in the workplace (β = -0.157, *p* < 0.001), an expectation of better health (β = 0.133, *p* < 0.001), health stressor (β = 0.076, *p* < 0.001), state of health (β = 0.090, *p* < 0.001), feeling of tension, stiffness, or lack of flexibility in the spine (β = 0.041, *p* = 0.004), ideal body weight for height (β = 0.035, *p* = 0.017), difficulty thinking, concentrating, or indecisiveness (β = 0.050, *p* = 0.001), ability to cope with daily problems (β = 0.034, *p* = 0.015), and frequent high fibre diet (β = 0.027, *p* = 0.039)
	Model summary: *F*(16, 540) = 350.45, *p* < 0.001*, R = 0.760, Adjusted R^**2**^** = 0.919**
**(2). General health condition**	(Constant), feeling spinal tension (β = 0.123, *p* < 0.001), stiffness or lack of flexibility (β = 0.117, *p* < 0.001), dizziness or light-headedness (β = 0.122, *p* < 0.001), sore limb (β = 0.153, *p* < 0.001), allergies or skin diseases (β = 0.109, *p* < 0.001), fatigue or low energy (β = 0.117, *p* < 0.001), being hypertensive (β = 0.124, *p* < 0.001), asthmatic (β = 0.121, *p* < 0.001), diabetes (β = 0.093, *p* < 0.001), incidences of headaches, chest pain, nausea, or abdominal discomfort (β = 0.090, *p* < 0.001), neck or backache (β = 0.078, *p* < 0.001), breathless with slight exertion (β = 0.117, *p* < 0.001), colds, flu or cough (β = 0.087, *p* < 0.001), history of accidents (β = 0.080, *p* < 0.001), falling or tripping (β = 0.077, *p* < 0.001), feeling limited by physical health at work (β = -0.040, *p* < 0.001), being free of physical pain (β = 0.044, *p* = 0.001), presence of heart disease (β = 0.053, *p* < 0.001), sleep free of bad dreams (β = 0.040, *p* < 0.001), concern about general status (β = -0.041, *p* < 0.001), always ate high fibres diet (β = 0.049, *p* < 0.001), job-related burnout (β = -0.048, *p* < 0.001), alcoholic (β = -0.026, *p* = 0.019), and willingness to recommend LTC job to others (β = -0.032, *p* = 0.007)
	Model summary: *F*(24, 532) = 350.45, p < 0.001*, R = 0.921, Adjusted R^**2**^** = 0.919**
**(3). Mental and emotional health**	(Constant), peace of mind (β = 0.071, *p* < 0.001), depression (β = -0.065, *p* = 0.002), job-related burnout (β = -0.084, *p* < 0.001), personal accomplishment (β = 0.049, *p* = 0.003), insomnia (β = -0.105, *p* < 0.001), mental health (β = 0.066, *p* < 0.001), moodiness, temper, or angry outbursts (β = -0.094, *p* < 0.001), feeling that work concerns would be addressed (β = 0.083, *p* < 0.001), sleep free of bad dreams (β = 0.060, *p* = 0.001), health-related quality of life (β = 0.063, *p* < 0.001), feeling delighted with job accomplishments (β = 0.061, *p* = 0.001), being worried about small things (β = 0.068, *p* < 0.001), tight work schedule (β = -0.073, *p* < 0.001), sense of belonging in workplace (β = -0.063, *p* < 0.001), anxiety (β = 0.057, *p* = 0.001), stress free (β = -0.044, *p* = 0.014), incidence of colds (β = 0.070, *p* < 0.001), flu or cough (β = 0.039, *p* = 0.004), negative or critical feelings about one’s self (β = -0.042, *p* = 0.015), having no control over one’s work (β = -0.067, *p* < 0.001), unexplained sadness (β = -0.062, *p* = 0.001), good level of motivation (β = 0.052, *p* = 0.001), willingness to recommend LTC job to others (β = 0.034, *p* = 0.029), being fidgety or restless (β = -0.057, *p* < 0.001), work-related quality of life (β = 0.039, *p* = 0.014), health-related stress (β = 0.046, *p* = 0.003), depressed at work (β = -0.048, *p* = 0.004), absence due to work-related injury (β = 0.029, *p* = 0.020), getting recommended level of exercise (β = -0.035, *p* = 0.008), being comfortable with ‘negative’ emotions (β = -0.039, *p* = 0.004), being hypertensive (β = -0.028, *p* = 0.031), breathless with slight exertion (β = 0.033, *p* = 0.021), ability to handle fear and anxiety (β = 0.051, *p* = 0.003), excited to be alive (β = 0.036, *p* = 0.017), overall satisfaction with my health (β = -0.048, *p* = 0.006), and incidence of nausea or abdominal discomfort (β = 0.032, *p* = 0.025)
	Model summary: *F*(36, 520) = 163.04, *p* < 0.001*, R = 0.927, Adjusted R^**2**^** = 0.921**
**(4). Stress level**	(Constant), emotional stress (β = 0.112, *p* < 0.001), financial stress (β = 0.230, *p* < 0.001), relationship stress (β = 0.155, *p* < 0.001), stress from sex life (β = 0.162, *p* < 0.001), work stress (β = 0.101, *p* < 0.001), academic stress (β = 0.141, *p* < 0.001), family stress (β = 0.121, *p* < 0.001), unexplained sadness (β = -0.033, *p* = 0.029), coping with daily problems (β = 0.084, *p* < 0.001), health stressors (β = 0.060, *p* < 0.001), quality of personal life (β = 0.050, *p* = 0.005), feeling limited by physical health at work (β = -0.039, *p* = 0.004), concern about general status (β = 0.100, *p* < 0.001), emotional health (β = 0.039, *p* = 0.006), tight work schedule (β = -0.044, *p* = 0.002), I seem to get sick a little easier than other people (β = 0.040, *p* = 0.004), I am healthy as anybody I know (β = -0.037, *p* = 0.011), quality of romantic life (β = 0.048, *p* = 0.005), believe that work concerns would be addressed (β = 0.033, *p* = 0.018), I am free of chronic disease (β = 0.033, *p* = 0.020), and getting recommended level of exercise (β = 0.029, *p* = 0.039)
	Model summary: *F*(21, 535) = 270.09, *p* < 0.001*, R = 0.960 Adjusted R^**2**^** = 0.918**
**(5). Quality of life**	(Constant), quality of life as a whole (β = 0.116, *p* < 0.001), quality of romantic life (β = 0.149, *p* < 0.001), personal accomplishment (β = 0.188, *p* < 0.001), financial needs (β = 0.150, *p* < 0.001), health-related quality of life (β = 0.132, *p* < 0.001), work-related quality of life (β = 0.122, *p* < 0.001), quality of personal life (β = 0.119, *p* < 0.001), teamwork (β = 0.124, *p* < 0.001), physical appearance (β = 0.140, *p* < 0.001), quality of relationship life (β = 0.140, *p* < 0.001), handling of problems (β = 0.110, *p* < 0.001), job-related burnout (β = 0.000, *p* = 0.006), presence of heart disease (β = 0.000, *p* < 0.001), having chest pain (β = 0.000, *p* < 0.001), compassion for work (β = 0.000, *p* = 0.002), ability to handle fear and anxiety (β = 0.000, *p* = 0.001), sleep free of bad dreams (β = 0.000, *p* = 0.006), sense of belonging in the workplace (β = 0.000, *p* = 0.002), believe that work concerns would be addressed (β = 0.000, *p* = 0.009), allergies or skin diseases (β = 0.000, *p* = 0.042), feeling delighted with job accomplishments (β = 0.000, *p* = 0.033), routine medical check-up (β = 0.000, *p* = 0.046), adherence to Canadian food guide (β = 0.000, *p* = 0.008), and getting enough sleep (β = 0.000, *p* = 0.037)
	Model summary: *F*(24, 532) = 194.13, *p* < 0.001*, R = 0.999, Adjusted R^**2**^** = 0.998**
**(6). Health behaviour**	(Constant), healthy diet (β = 0.105, *p* < 0.001), relaxation (β = 0.135, *p* < 0.001), adherence to the Canadian food guide (β = 0.114, *p* < 0.001), cancer screening (β = 0.138, *p* < 0.001), smoking (β = 0.152, *p* < 0.001), ideal body weight for height (β = 0.163, *p* < 0.001), always ate diets high in fibre (β = 0.108, *p* < 0.001), routine medical check-ups (β = 0.145, *p* < 0.001), getting recommended level of exercise (β = 0.174, *p* < 0.001), alcoholic (β = 0.135, *p* < 0.001), getting enough sleep (β = 0.109, *p* < 0.001), getting health counselling (β = 0.156, *p* < 0.001), drinking an adequate amount of fluid (β = 0.112, *p* < 0.001), avoidance of high-fat foods (β = 0.109, *p* < 0.001), being health conscious (β = 0.80, *p* < 0.001), excited to be alive (β = 0.033, *p* = 0.008), presence of neck or back aches (β = 0.037, *p* = 0.005), contemplation to quit job or profession (β = 0.025, *p* = 0.039), anxiety (β = 0.027, *p* = 0.045), fatigue or low energy (β = 0.035, *p* = 0.012), incidence of headaches (β = -0.033, *p* = 0.010), insomnia (β = -0.030, *p* = 0.022), and incidence of colds, flu or cough (β = 0.026, *p* = 0.030)
	Model summary: *F*(23, 533) = 312.72, *p* < 0.001*, R = 0.969, Adjusted R^**2**^** = 0.936**
**(7). Turnover and Absenteeism**	(Constant), contemplation to change employer or workplace (β = 0.425, *p* < 0.001), absence due to ill-health (β = 0.285, *p* < 0.001), contemplation to quit job or profession (β = 0.429, p < 0.001), work stress (β = 0.235, *p* < 0.001), and absence due to work-related injury (β = 0.485, *p* < 0.001)
	Model summary: *F*(5, 551) = 76.62, *p* < 0.001*, R = 0.999, Adjusted R^**2**^** = 0.998**

^*^β (beta = standardized regression coefficient) and F statistic were significant at *p* < 0.05 (2-tailed)

## Discussion

The population of older adults in Canada is increasing [1], who will eventually need continuing care and the services of professional caregivers [3]. Consequently, the Government of Canada has been responding to the anticipated surge in the population of older adults by making policies and collaborating with continuing care facilities to expand their capacities and the services they can provide [6, 10, 24]. This study was a pre-Covid-19 pandemic survey, but the unprecedented LTC and AL staff shortages following the pandemic have added more relevance to our findings. The need to care for the vulnerable in our society is well recognised, but the question rarely asked is, who cares for caregivers? Our study was grounded in the “happy-productive worker” theory, which states that workers are biopsychosocial beings whose conducive work environment and well-being are positive determinants of individual and organisational performances [23]. Therefore, we investigated the health status, stress level, QOL, absenteeism, and the tendency of organisational and professional turnover among Alberta’s LTC and AL caregivers.

About half of the participants were 45 years or older. It can be projected that by the next two decades, these caregivers will turn to older adults who need care themselves. Coupled with Alberta’s aging population likely to double within the same period, the problematic shortage of continuing care workforce will compound [3]. A strategic intervention for continuing care workforce is warranted.

The majority of caregivers in our study were females. This finding concurred with that of other researchers who reported that females predominantly provided long-term caregiving services [9, 32]. Similarly, data from the Organisation for Economic Cooperation and Development countries showed that women held on average 90% of the jobs in the LTC sector [33]. Caregiving involves significant mental and physical stress, especially when caring for older adults who cannot independently carry out the basic activities of daily living [34, 35]. Caregivers are often required to lift, transfer, and transport older adults who have severe mobility disabilities or to provide them with ambulatory assistance. Josephson et al. [36] opined that work-related musculoskeletal disorder in women is complicated by female physiology and other family roles such as home upkeep, and childcare. Bearing in mind that women are additionally stressed by family roles, Oluka et al. [37] recommended a flexible work schedule with shortened shift duration in facilities predominated by women of childbearing age. Therefore, facility administrators should employ more staff, and improve workers’ welfare in a way to attract and retain an adequate workforce that comprises both genders [32].

Going by the recommendations of other researchers that job schedules should be flexible to accommodate personal and family responsibilities for women in particular [25, 37], we expected a lesser workload for our study participants. On the contrary, our findings showed that about a quarter of the participants worked more than the standard working hours in Canada (40 h per week). Holroyd-Leduc and Laupacis [9] noted that some Canadian caregivers earn as little as $14 per hour. Consequently, many caregivers take multiple jobs across available shifts in different facilities to meet their financial obligations, as well as avoid working overtime in a particular facility [7]. A strained workforce impacts the quality of care for clients and the health status of caregivers [24]. Anecdotally, work overload among caregivers can be elective (for personal economic reasons) or obligatory (to make up for staff shortages). Desimini [25] revealed that staff shortages are often associated with multiple shifts. Thus, the multiple shifts reported in our study could be extrapolated as a cohort of underpaid caregivers working across understaffed facilities.

The need to improve the welfare of caregivers cannot be overemphasised as it has important public health and economic implications [38]. The roles of caregiving pose significant occupational health hazards that can affect caregivers’ abilities to maintain good physical and general health [39]. Nonetheless, the majority of our study participants reported that they were satisfied with their overall health conditions. Very few caregivers reported poor physical condition as compared with people of their age. Grossman and Webb [40] suggested that occupational health hazards are reported to be more profound in older caregivers. In our study, over two-thirds of caregivers reported fatigue or low energy as a significant physical health complaint. This outcome was consistent with the study of Harris [41], who found that caregivers were mostly fatigued more than they can perceive. This might be a physical health implication of work overload among this cohort. Improved workplace ergonomics, exercise, and leisure time have been recommended to mitigate physical health issues among continuing caregivers [25]. Therefore, LTC and AL facilities should include a fitness room or outdoor games facilities to encourage residents' and caregivers’ participation in community fitness programmes [42].

There was a moderate correlation between stress levels, health behaviours, mental and emotional health, and QOL. Caring for institutionalised older adults is very stressful due to their often vulnerable physical, physiological, and psychological state. This entails caring with care, endurance, and dedication. Caregivers for older adults often suffer work-related stress, depression, frustration, and physical health effects [43]. Our study participants reported that they experienced high levels of stress coping with work, relationships, financial, and family problems. Previous studies have found a positive correlation between workload and occupational stress [34, 44, 45]. The high stress levels among our study participants could have impacted their health behaviours, for instance, we found that 55.2% of the participants used alcohol, perhaps as a stress-coping strategy. The second and sixth regression models showed that health status and health behaviour were significantly associated with alcohol use. However, we did not obtain information on participants' intent, frequency, and quantity of alcohol intake.

There was a correlation between poor mental health and turnover/absenteeism. Addressing the situations that expose caregivers to poor mental health may mitigate their turnover and absenteeism. Zacharopoulou et al. [35] reviewed several studies and found that caregivers were at great risk for developing major emotional disorders such as depression and anxiety. In contrast to Zacharopoulou et al. [35], only 21.3% of our caregivers reported that they experienced vague fears or anxiety. The majority of caregivers surveyed reported satisfactory mental and emotional health as well as healthy living behaviours, including adherence to the Canadian Food Guide (CFG) [46]. The CFG recommended adequate fluid intake, avoidance of smoking and acute health problems [46]. We also noted that some facilities in our survey had implemented the Mental Health Commission of Canada’s guidelines [47] which recommended better strategies to support caregivers in maintaining their mental and physical well-being. However, more collaborative efforts are still needed to address the physical and mental health challenges among caregivers [9, 11, 12].

The challenges caregivers face while discharging their duties are likely the main precursor of their poor QOL perception [48]. Caregivers’ perception of their QOL has been linked to the quality of their service delivery [25, 45, 49]. We found that three-quarters of our participants were at least satisfied with their QOL, and while this finding is encouraging, we are concerned about the negative impact of poor QOL on the services rendered by the other one-quarter of caregivers. We found a moderate association between QOL, stress level, and turnover/absenteeism. Increased stress levels may result in poor QOL, physical health, and absenteeism, thus exposing other caregivers to higher workloads, dissatisfaction, and ultimately increase organisational turnover [50, 51]. Nonetheless, 22.3% who were not satisfied with their jobs are particularly important given the direct relationship between job satisfaction and intent to quit [13, 51, 52].

Finally, one in every six caregivers reported being absent from work for six days or more in the last six months due to ill health or work-related injuries. Facilities should pay particular attention to any recurrent absenteeism among their staff, for the root cause to be investigated to mitigate organisational turnover [12, 50]. About half of the participants had contemplated quitting the caregiving profession or changing the facility at which they worked. Goins [53] reported a high turnover among caregivers in LTC facilities. The high turnover rate has been linked to higher organisational costs, resulting in decreased productivity and quality of care among LTC and AL facilities [21, 51, 53].

### Implication for practice

Historically, Canadian society has undervalued the continuing care workforce, which consists mostly of underpaid women working in understaffed facilities [9]. Findings from the current study suggest that caregivers’ poor well-being could lead to high staff turnover and absenteeism in the sector. Therefore, we recommend designing policies that can improve caregivers’ physical and mental health, quality of life, and well-being to improve caregiver retention. Such strategies include adequate staffing based on a predetermined staff-to-client ratio, improved wages, shorter and flexible shift duration, allowing more casual leaves, annual vacation, adequate sleep time, exercising, and socialisation within the facility. Continuing care organisations should avail their workers of robust health coverage including mental health counselling. Moreover, managers should pursue a safe work environment including strategies for housekeeping to reduce the incidence of workplace injury, and the provision of assistive work tools such as laundry baskets with wheels and bed roller sheets to reduce manual handling [25, 42]. These recommendations have become more germane with the further negative impact of the Covid-19 pandemic on the sector [2, 17, 19].

### Limitations

The strength of our study lies in the use of questions from standardised instruments and obtaining proportional representation from all five Alberta Health Zones. We felt that there were some limitations in the recruitment process. Convenience sampling was used to select individual participants within each facility which can lead to nonresponse bias. As with most survey designs, we relied on self-reported information, which has its limitations because authors are unable to directly determine the veracity of the reports. Hence, we cannot ascertain causal relationships. Although one-way ANOVA is a robust statistical tool for detecting mean differences between independent samples with unequal sample sizes, the reader should note that the descriptive statistics were generated from a cohort dominated by Health Care Aides (58.1%).

## Conclusion

A greater percentage of the caregivers in our survey reported good health conditions, including their, QOL, physical, mental, emotional, and behavioural health status. However, we found that both workload and stress levels were high, which are likely leading to occasional absenteeism, job dissatisfaction, and intention to quit. The danger of unchecked organisational and professional turnover among Albertan LTC and AL caregivers is obvious. As a greater percentage of the Canadian population continue to age, more professional caregivers will be needed for older adults. We think that the anticipated shortage of caregivers can be mitigated by improving the well-being of caregivers as we recommended.

## Supplementary Information

**Additional file 1:** **Supplementary file 1. **Tabular presentations of mental health, stress, and quality of life levels.

## Data Availability

The datasets analysed during the current study are available from the corresponding authors on reasonable request. The dataset could not be anonymised enough for public release.

## Abbreviations

AHS: Alberta Health Services
AHW: Allied Health Workers
AL: Assisted Living
CFG: Canadian Food Guide
GHC: General Health Condition
HCA: Health Care Aide
LPN: Licenced Practical Nurse
LTC: Long-Term Care
MEH: Mental and Emotional Health
PH: Physical Health
QOL: Quality of Life
RN: Registered Nurse
TAA: Turnover and Absenteeism
