# Supplementary material for: Well-being of professional older adults’ caregivers in Alberta’s assisted living and long-term care facilities: a cross-sectional study
Source: BMC Geriatr. 2023 Feb 9;23:85. doi: 10.1186/s12877-023-03801-9 (PMC9908505; doi:10.1186/s12877-023-03801-9)
Supplement: Supplementary file 1 — Additional file 1: Supplementary file 1. Tabular presentations of mental health, stress, and quality of life levels. [file 12877_2023_3801_MOESM1_ESM.docx]

**Supplementary file 1** Tabular presentations of mental health, stress, and quality of life levels.

Mental and Emotional Health of Caregivers

| Think about the last six months and answer the following questions: | Strongly Agree  *f* (%) | Agree  *f* (%) | Neutral  *f* (%) | Disagree  *f* (%) | Strongly Disagree *f* (%) |
| --- | --- | --- | --- | --- | --- |
| I felt depressed or sad much of the time in the last six months (n=932) | 37(4.0) | 160(17.2) | 189(20.3) | 356(38.2) | 190(20.4) |
| I have negative or critical feelings about myself (n=929) | 22(2.4) | 164(17.7) | 198(21.3) | 364(39.2) | 181(19.5) |
| I experience moodiness, temper, or angry outbursts (n=927) | 41(4.4) | 252(27.2) | 174(18.8) | 320(34.5) | 140(15.1) |
| I experience depression or lack of interest (n=930) | 47(5.1) | 207(22.3) | 158(17.0) | 323(34.7) | 195(21.0) |
| I am more worried about small things (n=929) | 28(3.0) | 187(20.1) | 215(23.1) | 354(38.1) | 145(15.6) |
| I have difficulty thinking, concentrating, or indecisiveness (n=928) | 22(2.4) | 135(14.5) | 171(18.4) | 412(44.4) | 188(20.3) |
| I experience vague fears or anxiety (n=929) | 29(3.1) | 169(18.2) | 180(19.4) | 360(38.8) | 191(20.6) |
| I am fidgety or restless (n=927) | 21(2.3) | 110(11.9) | 164(17.7) | 412(44.4) | 220(23.7) |
| I have difficulty falling or staying asleep (n=927) | 111(12.0) | 215(23.2) | 162(17.5) | 277(29.9) | 162(17.5) |
| I experience recurring thoughts or dreams (n=918) | 24(2.6) | 130(14.2) | 158(17.2) | 380(41.4) | 226(24.6) |
| I do not have time to do the work that must be done (n=929) | 99(10.7) | 181(19.5) | 183(19.7) | 324(34.9) | 142(15.3) |
| I have no control over how I do my work (n=925) | 147(15.9) | 334(36.1) | 149(16.1) | 217(23.5) | 78(8.4) |
| I feel depressed at work (n=926) | 20(2.2) | 71(7.7) | 155(16.7) | 418(45.1) | 262(28.3) |
| I feel delighted when I accomplish something at work (n=929) | 248(26.7) | 478(51.5) | 119(12.8) | 51(5.5) | 33(3.6) |
| I feel burned out from my work (n=928) | 96(10.3) | 200(21.6) | 281(30.3) | 244(26.3) | 107(11.5) |
| I am mentally healthy (n=931) | 237(25.5) | 395(42.4) | 197(21.2) | 59(6.3) | 43(4.6) |
| I am emotionally healthy (n=931) | 219(23.5) | 393(42.2) | 207(22.2) | 73(7.8) | 39(4.2) |

Evaluation of the Stress Levels of Caregivers

| Evaluate your stress relative to the following: | None  *f* (%) | Low  *f* (%) | Medium *f* *f* (%) | High  *f* (%) | Very High *f* (%) |
| --- | --- | --- | --- | --- | --- |
| Family (n=928) | 156 (16.8) | 404 (43.5) | 243 (26.2) | 104 (11.2) | 21 (2.3) |
| Work (n=925) | 57 (6.2) | 267 (28.9) | 354 (38.3) | 156 (16.9) | 91 (9.8) |
| Significant relationship: spouse or partner (n=916) | 315 (34.4) | 360 (39.3) | 158 (17.2) | 58 (6.3) | 25 (2.7) |
| Health (n=927) | 213 (23.0) | 421 (45.4) | 222 (23.9) | 55 (5.9) | 16 (1.7) |
| Finance (n=924) | 143 (15.5) | 283 (30.6) | 283 (30.6) | 131 (14.2) | 84 (9.1) |
| Sex life (n=916) | 470 (51.3) | 285 (31.1) | 109 (11.9) | 33 (3.6) | 19 (2.1) |
| School (n=897) | 687 (76.6) | 113 (12.6) | 53 (5.9) | 30 (3.3) | 14 (1.6) |
| General status (n=923) | 292 (31.6) | 402 (43.6) | 203 (22.0) | 19 (2.1) | 7 (0.8) |
| Emotional status (n=928) | 228 (24.6) | 415 (44.7) | 226 (24.4) | 48 (5.2) | 11 (1.2) |
| Coping with daily problems  (n=931) | 178 (19.1) | 473 (50.8) | 230 (24.7) | 38 (4.1) | 12 (1.3) |

Evaluation of the Quality of Life of Caregivers

| Evaluate your feeling regarding the quality of your life with: | Happy  *f* (%) | Satisfied  *f* (%) | Mixed  *f* (%) | Unhappy  *f* (%) | Terrible  *f* (%) |
| --- | --- | --- | --- | --- | --- |
| Your personal life (n=930) | 459 (49.4) | 283 (30.4) | 170 (18.3) | 16 (1.7) | 2 (0.2) |
| Your significant other/ spouse/ partner (n=881) | 503 (57.1) | 198 (22.5) | 136 (15.4) | 29 (3.3) | 15 (1.7) |
| Your romantic life (n=900) | 429 (47.7) | 234 (26.0) | 154 (17.1) | 60 (6.7) | 23 (2.6) |
| Your financial needs (n=932) | 202 (21.7) | 334 (35.8) | 257 (27.6) | 108 (11.6) | 31 (3.3) |
| Your co-workers (n=929) | 203 (21.9) | 347 (37.4) | 325 (35.0) | 39 (4.2) | 15 (1.6) |
| The actual work you do your job (n=930) | 328 (35.3) | 392 (42.2) | 176 (18.9) | 25 (2.7) | 9 (1.0) |
| Your handling of problems in your life (n=923) | 179 (19.4) | 455 (49.3) | 256 (27.7) | 29 (3.1) | 4 (0.4) |
| What you are actually accomplishing (n=929) | 257 (27.7) | 407 (43.8) | 230 (24.8) | 32 (3.4) | 3 (0.3) |
| Your physical appearance (n=932) | 231 (24.8) | 368 (39.5) | 238 (25.5) | 74 (7.9) | 21 (2.3) |
| Your health (n=929) | 268 (28.8) | 368 (39.6) | 235 (25.3) | 49 (5.3) | 9 (1.0) |
| Your life as a whole (n=931) | 342 (36.7) | 375 (40.3) | 194 (20.8) | 19 (2.0) | 1 (0.1) |
